# Wall Materials for Encapsulating Bioactive Compounds via Spray-Drying: A Review

**DOI:** 10.3390/polym15122659

**Published:** 2023-06-12

**Authors:** Elsa Díaz-Montes

**Affiliations:** Unidad Profesional Interdisciplinaria de Biotecnología, Instituto Politécnico Nacional, Av. Acueducto s/n, Barrio La Laguna Ticoman, Ciudad de Mexico 07340, Mexico; elsadimo123@gmail.com

**Keywords:** wall material, biopolymers, carbohydrates, gums, proteins, lipids, spray-drying, encapsulation efficiency

## Abstract

Spray-drying is a continuous encapsulation method that effectively preserves, stabilizes, and retards the degradation of bioactive compounds by encapsulating them within a wall material. The resulting capsules exhibit diverse characteristics influenced by factors such as operating conditions (e.g., air temperature and feed rate) and the interactions between the bioactive compounds and the wall material. This review aims to compile recent research (within the past 5 years) on spray-drying for bioactive compound encapsulation, emphasizing the significance of wall materials in spray-drying and their impact on encapsulation yield, efficiency, and capsule morphology.

## 1. Introduction

Encapsulation is a process that involves trapping a particle, substance, or compound (core material) within a material called the wall material [1]. Encapsulation has been widely employed in various industries to protect, stabilize, and/or delay the degradation of components [2]. For instance, in the food industry, it is utilized to preserve enzymes, flavors, colors, and aromas, enhancing their stability, improving textures, and enabling controlled release [3]. In the pharmaceutical industry, it is used to facilitate easy administration and rapid absorption of oral drugs [4]. Meanwhile, the cosmetics industry employs encapsulation to develop personal care products such as anti-aging creams, sunscreens, moisturizers, and fragrances, which contain active substances (e.g., antioxidants, sunscreens, and whitening agents) [5].

**Figure 1:**
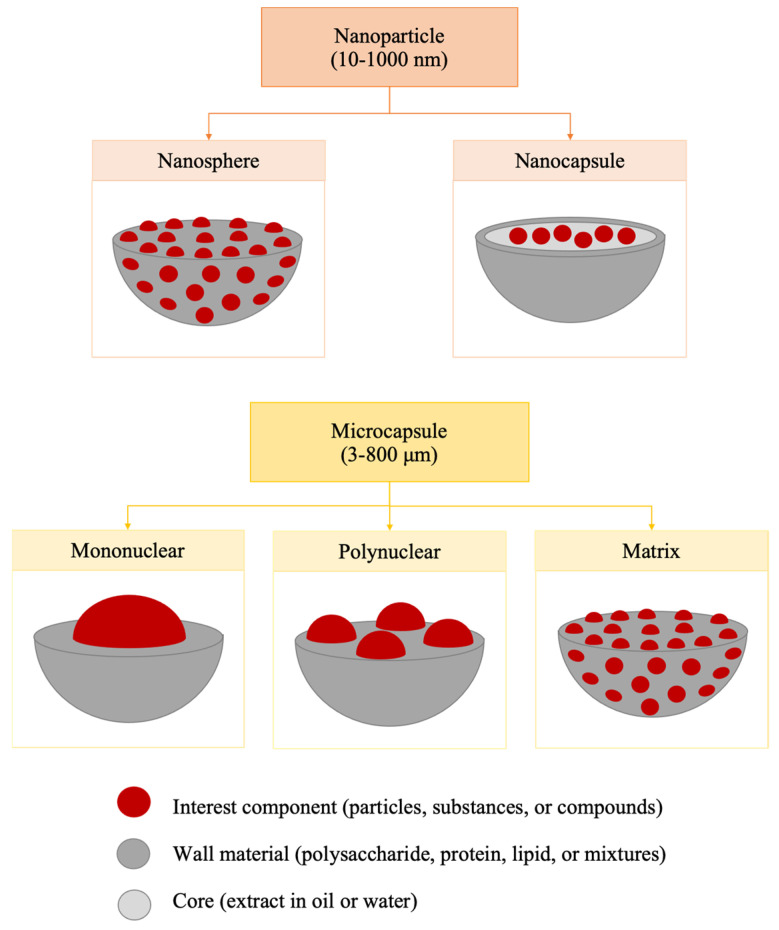
Types of nanoparticles and microcapsules.

Encapsulation processes can be carried out using hot techniques (e.g., solvent evaporation, spray-drying, and melt extrusion) or cold techniques (e.g., spray-chilling and freeze-drying), with spray-drying being the most widely used technique at the industrial level [6]. Depending on the technique, nanoparticles (capsules or spheres) ranging from 10 to 1000 nm or microcapsules (mononuclear, multinuclear, or matrix) from 3 to 800 µm can be generated [7,8], as depicted in Figure 1.

Nanocapsules are systems in which the compound of interest is surrounded by a single polymer, while nanospheres have the compound uniformly dispersed within their matrix [9]. Mononuclear microspheres have the compound surrounded by the wall material, multinuclear microspheres have multiple compounds trapped in their core, and matrix-type microspheres have the compound distributed homogeneously throughout the wall material [8].

The most commonly used wall materials in encapsulation processes include carbohydrates (such as maltodextrin, starch, dextran, alginate, chitosan, and gums), gums (such as Arabic, karaya, and xanthan), fibers (such as pectin and carrageenan), proteins (such as whey, casein, and gelatin), and waxes (such as beeswax, carnauba, and candelilla) [10].

**Figure 2:**
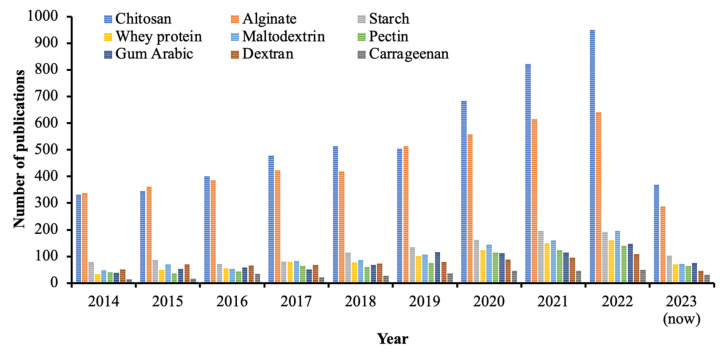
Number of publications related to encapsulation processes using various wall materials (source: *Scopus*; keywords: encapsulation, chitosan, alginate, starch, whey protein, maltodextrin, pectin, gum Arabic, dextran, and carrageenan; accessed: 23 May 2023).

Figure 2 illustrates publications from the last decade that are related to the wall materials used in encapsulation processes. The wall material plays a crucial role in encapsulation processes, and its selection often involves a trial-and-error approach. However, this approach can result in time and resource losses. The most viable option for choosing wall materials is to consider the compatibility of their physicochemical characteristics with the compounds to be encapsulated.

Based on the information provided, the aim of this study was to gather and summarize general information about encapsulation processes and the most commonly used wall materials in the industry. Relevant information (within the last 5 years) related to the spray-drying of bioactive components using various wall materials was analyzed.

**Figure 3:**
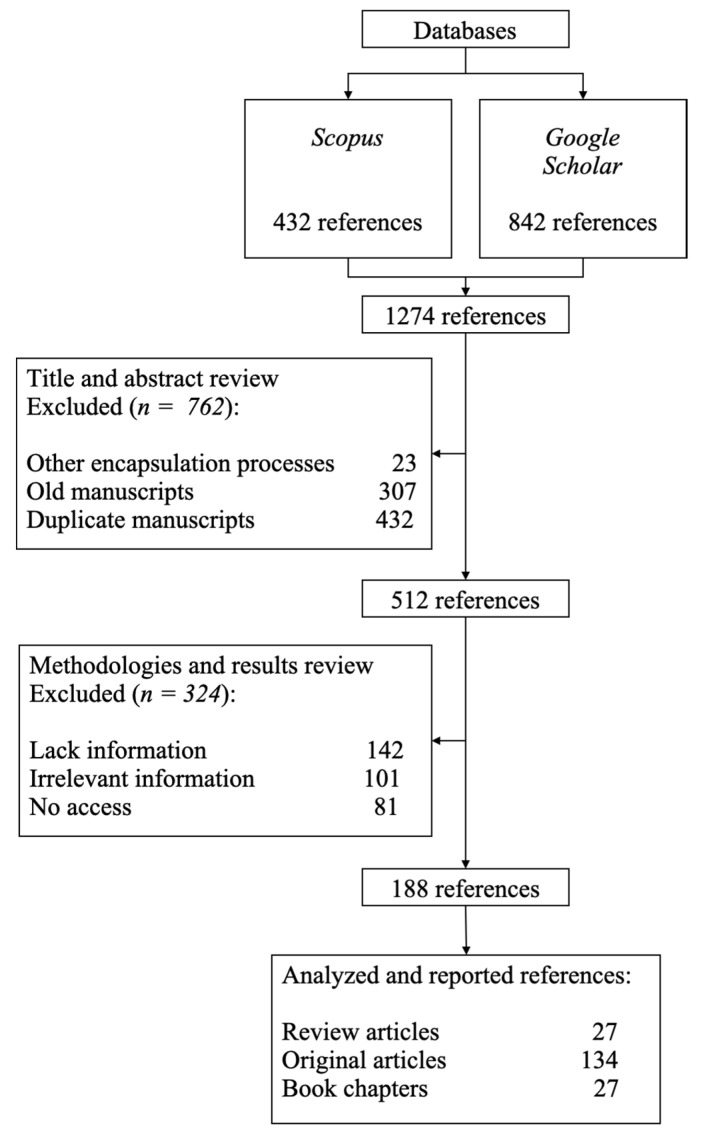
Flowchart of the references selected for the systematic review.

The bibliographic search was conducted from 6 November 2021, to 23 May 2023, using the Scopus and Google Scholar databases. The search focused on the following keywords: encapsulation, spray-drying, chitosan, whey protein, gum Arabic, alginate, maltodextrin, dextran, starch, pectin, and carrageenan. Three types of documents were sought: book chapters, review articles, and original research articles. Book chapters and review articles were selected if the title contained the word “encapsulation”, regardless of the publication year. Original research articles had to meet two criteria: (1) being published between 2017 and 2023, and (2) mentioning spray-drying of bioactive compounds in the title or abstract. The selected information was then used to gather introductory information, definitions, contextualization, and a general overview from the review articles and book chapters. For the discussion, the information was synthesized from the methodologies and most relevant findings of the original research articles. Figure 3 summarizes the search strategy and the inclusion/exclusion criteria of the bibliographic search. The selected information was synthesized into images and tables for analysis and discussion in the subsequent sections.

## 2. Spray-Drying Process

Among the various encapsulation processes, techniques such as spray-drying, spray bed drying, fluid bed coating, and spray-cooling are employed, each with its own mode of operation, although they share the common goal of producing dry particles [11]. Spray-drying, in particular, is a unit process that converts a liquid dispersion into dry particles (<40 µm) and is considered one of the oldest and most widely used encapsulation techniques. It is renowned for its simplicity, user-friendliness, speed, continuous operation, and cost-effectiveness [2,12]. It is estimated that approximately 90% of encapsulated products, including ingredients and additives, are manufactured using this process [12].

### Stages of the Spray-Drying Process

**Figure 4:**
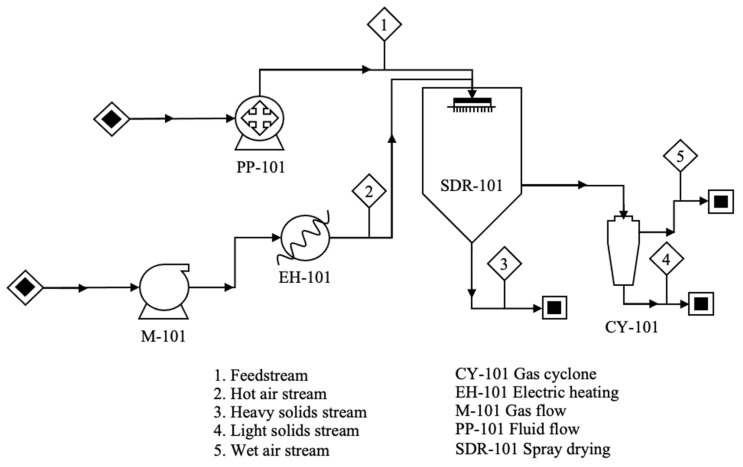
Schematic diagram of the spray-drying process.

The spray-drying process is conducted using equipment such as the one depicted in Figure 4, and involves five consecutive stages. First, a liquid solution (without wall material) or a liquid dispersion (containing wall material) is sprayed through an atomizer, which can be pneumatic, rotating disk, fluid nozzle, pressure nozzle, or sonic nozzle [5]. Second, the generated droplets descend through the vacuum chamber, where hot gas (typically air) circulates. Third, temperature differences facilitate the transfer of mass from liquid to gas. Fourth, the liquid exits the equipment in vapor form. Finally, the remaining solid from the dispersion is collected in a container [13]. The spray-drying process predominantly produces polynuclear or matrix microcapsules (as shown in Figure 1) [11]. However, their shape and morphology are influenced by factors such as wall material, the concentration of dispersion (ratio of wall material to core material), and the operating conditions of the dryer (such as air temperature and feed rate) [8,11].

## 3. Wall Materials Used in Spray-Drying

The wall material refers to the protective matrix that safeguards the core material, such as particles, substances, or compounds, throughout the encapsulation process and subsequent handling. It should possess the ability to withstand mechanical stress (e.g., handling) and environmental conditions (e.g., humidity, temperature, and water activity) [14]. In spray-drying processes, the chosen wall material must ensure the stability and shelf-life of the encapsulated particle, substance, or compound, while also being cost-effective in terms of encapsulation yield and efficiency [15]. It is essential to understand the characteristics of the materials, regardless of this section aiming to define the primary materials utilized in spray-drying processes.

In this section, the composition and characteristics of the most commonly employed wall materials in spray-drying processes are described.

### 3.1. Polysaccharides

Polysaccharides are chains of simple sugars linked by glycosidic bonds. They are naturally synthesized by plants (e.g., starch and cellulose), animals (e.g., chitosan and chitin), and microorganisms (e.g., dextran and gellan gum) to produce energy and fulfill physiological and structural functions [16]. Some polysaccharides can also be enzymatically and chemically synthesized (e.g., condensation), such as certain cyclodextrins and chitosan derivates, to create non-natural, well-defined, and pure structures [17]. In commercial applications, polysaccharides are widely used as emulsifiers, gelling agents, flavorings, and encapsulants [18] as a result of their physicochemical properties such as viscosity and solubility [16]. They are commonly employed as ingredients in confectionery, beer, fried foods, ice creams, and sausages [18]. The most frequently used polysaccharides in spray-drying processes due to their low costs are starch, maltodextrin, chitosan, dextran, carrageenan, and gums [10]. The main characteristics of each are as follows:

- Starch is a complex polysaccharide composed of amylose and amylopectin, primarily derived from tubers (e.g., potatoes, cassava, and sweet potatoes) and cereals (e.g., corn, sorghum, wheat, rice, rye, oats, and barley) [19]. This carbohydrate is made up of glucose monomers with free hydroxyl groups (-OH) at positions C2, C3, and C6, giving it a highly hydrophilic helical structure [20]. Starch finds applications in various industries such as textiles, chemicals, healthcare, and food, due to its physicochemical properties such as solubility, viscosity, texture, and thermal stability [20].
- Maltodextrin is a polysaccharide derived from the hydrolysis of starch (from corn, rice, wheat, tapioca, sorghum, barley, etc.) with a dextrose equivalent value (DE: the ratio of reducing sugars to total sugars) of less than 20 [21]. Maltodextrin has different characteristics and properties compared to starch, leading to varied applications [22]. It is used as an additive in food products and beverages [23] and as a fat replacer in dairy, meat, and baked goods due to its ability to form gels, its hygroscopicity, solubility, viscosity, and sweetness [24].
- Chitosan is a structural polysaccharide extracted from microorganisms (such as fungi and algae), marine animals (such as crustaceans and mollusks), and insects (such as scorpions and spiders), or obtained through the chemical deacetylation of chitin [25]. Chitosan is highly regarded for its antibacterial, antifungal, and antiviral activity, attributed to its cationic polyelectrolyte character. It also possesses the ability to form gels due to its viscosity, plasticity, and solubility [26]. In recent years, chitosan has found applications in post-harvest pathogen control [27] and the development of biodegradable packaging [28].
- Dextran is a polysaccharide synthesized by microorganisms, particularly lactic acid bacteria, and it possesses various thermal, rheological, viscosity, and solubility properties due to its branching structure [29]. The application of dextran has been primarily explored as a food additive in the formulation of emulsions, nanoparticles, and immobilizers [30]. It is also used as an excipient in the formulation of inhaled drugs (such as rifampicin and budesonide) due to its humectant, stabilizing, and preserving action [31,32].
- Carrageenan is a sulfated polysaccharide extracted from red seaweeds such as *Kappaphycus* and *Eucheuma*. It exhibits structural diversity due to the degree of sulfation and can be classified as κ-, ι-, θ-, μ-, ν-, and λ-carrageenan [33]. Carrageenan does not have proven nutritional value, but it finds special application in the food industry due to its gelling, stabilizing, binding, and thickening properties. It is used in products such as jellies, dressings, fat substitutes, and pet food. Additionally, carrageenan has been utilized in experimental medicine, pharmaceuticals, and cosmetics as anti-inflammatory agents, hydrogels, drug carriers, and vehicle for drug delivery [34].
- Gums are water-soluble polysaccharides that do not have a specific classification but are recognized for producing viscous–sticky dispersions at low concentrations. Gums are extracted from algae (such as agars and alginates), microorganisms (such as gellan and xanthan), or higher plants (such as pectin, Arabic, and arabinogalactans) [35]. The gel-forming properties of gums are due to their affinity for water, allowing for rapid hydration and swelling of the structure. The degree of hydration results in various rheological properties that enable their application in construction materials (such as adhesives), food products (such as texture enhancers, stabilizers, and coatings), medical and pharmaceutical products (such as encapsulants), and textile products (such as additives) [36].

### 3.2. Proteins

Proteins are macromolecular structures composed of amino acids and play a vital role in all biological processes of living organisms. They can exist in the form of enzymes, hormones, antibodies, and receptors [37]. Proteins are part of the human diet as they are present in varying proportions in all animal- and plant-based products that are consumed. These macromolecules are valued in the industry for their gel-forming and foaming properties, particularly in the food industry, allowing their application to enrich existing products [38]. The use of proteins in spray-drying processes is feasible due to their ability to form rigid matrices, especially with proteins such as gluten, isolated proteins (e.g., soy and pea proteins), caseins, whey proteins, and gelatin [10]. Below, their main characteristics are described:

- Gluten is a mixture of insoluble, gummy proteins found in cereals such as wheat, rye, and barley. It is obtained by removing starch and soluble material from a dough made with grains [39]. The rheological properties of gluten facilitate the retention of air in the dough, making it particularly useful in processed food products such as breads, pasta, cookies, cakes, and other fermented goods [40].
- Casein is a group of proteins found in milk, which can be divided into four phosphoproteins: αS1, αS2, β, and κ-casein. These proteins organize themselves into micellar networks. Casein can be obtained through milk precipitation at pH 4.6, electrophoresis, or membrane processes [41]. The primarily significance of casein lies in the realm of sports, as it contributes to the nutritional composition of dietary supplements. However, it can also be applied in the formulation of nano and micro materials, food additives, and biodegradable films, as it can form gels when interacting with other polymers [42].
- Gelatin is a water-soluble protein derived from the hydrolysis of collagen, an insoluble product found in animal cartilage, skin, fibers, and tendons. Gelatin is classified as a hydrocolloid due to its high water-holding capacity. Its viscosity is its main property, which allows it to texture, thicken, stabilize emulsions, create foams, and form thermo-reversible gels [43]. Gelatin is free of sugars and fats and is rich in proteins. It is commonly used as an additive in food products such as confectionery, beverages, sweets, and dairy products. It also serves as an excipient in the pharmaceutical industry [44].
- Whey proteins are by-products obtained during the processing of dairy products such as cheese and casein. They can be classified into protein concentrates and protein isolates [45]. Whey proteins can be further categorized into four main proteins: β-lactoglobulin, α-lactalbumin, serum albumin, and immunoglobulin. Apart from their nutritional value, whey proteins possess binding and gelling properties, and they are capable of stabilizing foams and forming emulsions. As a result, they are used in various food products, including supplements, soups, sausages, desserts, and sweets) [46].

### 3.3. Lipids

Lipids are organic molecules that, as polysaccharides and proteins do, also play important biological and structural roles within living organisms. They are characterized by their insolubility in water [10]. Lipids exhibit a high degree of diversity due to their infinite structures; however, they can be generally classified into triacylglycerols, waxes esters, phosphoglycerides, sphingolipids, and sterols [47]. Lipids are used as fuels, plastics, detergents, soaps, paints, lubricants, and cosmetics. In the food industry, they are utilized as edible oils and coatings [48]. Among lipids, waxes are the most commonly employed in spray-drying encapsulation. Below, their main characteristics are described.

- Waxes are soft or sticky substances that form on the surface of plants (e.g., carnauba and candelilla), as well as on the body of animals (e.g., whales and sheep) and insects (e.g., bees). They are composed of long-chain aliphatic compounds that vary depending on their source of production, Waxes may contain fatty acids, primary and secondary alcohols, aldehydes, sterol esters, ketones, triacylglycerols, and triterpenes [49]. Waxes exhibit high hydrophobicity and resistance to hydrolytic degradation, making them suitable for use as protectants, surface polishes, lubricants, and repellents in the food, cosmetic and automotive industries [50].

## 4. Encapsulation of Bioactive Compounds Using Spray-Drying Processes

Bioactive compounds are secondary metabolites extracted from plants, and their significance lies in their wide range of properties that promote the health of humans and animals, such as antioxidant, antimicrobial, antibacterial, anti-inflammatory, and anticancer effects [51]. The most valued bioactive compounds for the industry include essential oils, carotenoids, fatty acids, phenolic acids, and flavonoids. These metabolites are extracted using methods such as solvent extraction, electrical pulses, hydrolysis, membrane systems, and others. However, the main challenge arises when it comes to their storage. Bioactive compounds can be volatile, thermolabile, and unstable, requiring protection to maintain their bioactive activity [52].

Spray-drying has emerged as a favorable alternative to protect bioactive compounds from environmental conditions, stabilize them, and enhance their bioavailability for potential application in food and pharmaceutical products. While there is no established protocol for conducting a spray-drying process for all bioactive compounds, there are five factors that can be considered:

- Functional groups of the wall material [53,54];
- Molecular weight of the wall material [55,56];
- Hydrophobicity/hydrophilicity of the wall material [57,58];
- Concentration of the wall material [59,60];
- Mixtures between wall materials [55,56];
- Operating conditions of the dryer [60,61].

These parameters significantly influence the stability of the encapsulates during the drying process and storage. This section summarizes studies from the last five years that have reported the use of spray-drying in the encapsulation of bioactive components from various plant sources. Tables are provided, which group the wall materials, operating conditions of the dryers, and the main results in terms of morphology, yield, efficacy, and preserved bioactive compounds.

### 4.1. Polysaccharide-Based Wall Materials

Polysaccharides are characterized by the presence of multiple -OH groups in their structure, enabling them to interact with each other and with other molecules, including water and other wall materials [62]. Based on Table 1, polysaccharides are widely employed as materials in spray-drying processes for encapsulating bioactive compounds. Here, some important findings in spray-drying with polysaccharides are presented.

- Starch is a carbohydrate that can undergo modifications to interact with hydrophilic and hydrophobic compounds. For example, Ocampo-Salinas et al. [72] stated that substituting the native groups of rice starch with octenyl-succinic anhydride modified its viscosity and thermal properties, enhancing its emulsifying capacity and facilitating the encapsulation process of bioactive compounds from vanilla. García-Gurrola et al. [115] modified starch through phosphorylation, esterification, and acetylation techniques, improving the retention and stability of encapsulated phenolic compounds extracted from red sorghum. This enhancement was attributed to increased hydration and swelling of the capsules. The study by García-Gurrola et al. [115] also demonstrated that starch acetylation increases its hydrophobic nature and improves the retention of lipidic bioactive compounds. Márquez-Gómez [70] reported that the mixture of native starch with modified starches (acetylated starch and maltodextrin) improved the stability and prevented the oxidation of essential orange oil. This improvement was attributed to a reduction in diffusivity and an increase in hydrophobicity through starch acetylation.
- Maltodextrin, a polysaccharide, plays a crucial role in encapsulation, particularly due to its DE level. The study carried out by Laokuldilok and Kanha [116] reports that as the DE decreased (from 30 to 10), the encapsulation efficiency of black rice anthocyanins increased by 30%. The authors observed that the increase in drying temperature also negatively impacted the encapsulation efficiency, but only in the encapsulates with a high DE, which could be attributed to increased oxidative reactions in the polymer. The effect of temperature in spray-drying processes with maltodextrin was also evaluated by Boyano-Orozco et al. [117], who found that the concentration of the wall material significantly affected the encapsulation efficiency when encapsulating phenolic compounds and tannins from rambutan peel. The authors noted that temperatures above 160 °C adversely affected the stability of the bioactive components when using maltodextrin concentrations below 10% *w/w*. Meanwhile, Balasubramani et al. [118] concluded, after encapsulating garlic oleoresin with maltodextrin, that the concentration of maltodextrin must be in an appropriate ratio to the core material concentration. Specifically, a wall material to core material ratio of 6:1 is required to ensure the highest encapsulation efficiency and component stability.
- Chitosan contains -OH groups at positions C3 and C6, as well as an amino group (-NH_2_) at position C2, which enables it to form ionic and electrostatic interactions with other molecules [119]. However, to achieve more rigid and resistant matrices, chitosan is often cross-linked with compounds that possess reactive functional groups, such as dialdehydes, glutaraldehyde, or tripolyphosphate [120]. For example, Aranaz et al. [102] encapsulated venlafaxine hydrochloride with chitosan obtained from two sources (blue crab and royal crab) and mixed it with tripolyphosphate. The authors observed that the degree of cross-linking between chitosan and tripolyphosphate varied among different experiments. Specifically, chitosan with higher viscosity exhibited less cross-linking, resulting in lower encapsulation efficiency. The study by Amorim et al. [121] reports that chitosan cross-linking improves with an increase in the inlet temperature during the spray-drying process.
- Dextran’s properties are primarily influenced by the molecular size of its chains. In a study by Wilson et al. [122], proteins were encapsulated with dextrans of two different sizes, 20 and 70 kDa, and it was observed that as the size increased, the available area also increased, resulting in a more rigid and less flexible three-dimensional structure. This improvement in structural properties enhanced the protein encapsulation efficiency. Another influential parameter in dextran encapsulation is temperature, as demonstrated by Wang and Meenach [123]. When encapsulating curcumin, the authors found that the highest encapsulation efficiency was achieved at a low dextran concentration (20%) and a high drying temperature (140 °C). The authors attributed these results to the polydispersity generated in the matrices, suggesting that the combination of low dextran concentration and high temperature contributed to the formation of more uniform and efficient encapsulation matrices.
- Carrageenan’s encapsulation efficiency is influenced by the type of component it encapsulates. Generally, any type of carrageenan is suitable for the encapsulating aqueous extracts. However, the study by Marín-Peñalver et al. [124] demonstrated that the encapsulation of lipid components is deficient. The interaction between carrageenan and lipids is very poor, resulting in incomplete homogenization and the components being left outside the capsules.
- Gums are another type of polysaccharide with gel-forming properties, which are attributed to their chemical structure consisting of -OH groups that may have branching or side substitutions of ester groups (-COO-R) or ether groups (ROR’), giving them a linear, helical, or cyclic conformation [36]. Gums undergo modifications during spray-drying processes, leading to the formation of encapsulates. Correâ-Filho et al. [60] encapsulated β-carotene with gum Arabic and evaluated the encapsulation yield at varying concentrations (5–35%) and temperatures (110–200 °C). The study reports that temperature influenced antioxidant activity only when the percentage of wall material was low, while the highest yield was obtained using intermediate levels of temperature and gum concentration. The morphology was affected by temperature, with lower temperatures resulting in microspheres with higher cavity content and rougher surfaces. Additionally, lower inlet temperatures resulted in smaller particles, which can be attributed to the increased swelling and shrinkage that occurs when water evaporates slowly.

Polysaccharides, including carbohydrates and gums, are highly versatile wall materials extensively used in encapsulation processes [125]. Their gelling properties enable the formation of matrices capable of entrapping various types of compounds. This ability is dependent on their chemical structure, the bonds they can establish with other molecules, their functional groups, and their molecular weight [126].

### 4.2. Protein-Based Wall Materials

Proteins possess a diverse array of functional groups, including carboxylic acids, amines, carboxamides, alcohols, thioethers, and thiols [127]. These functional groups enable proteins to interact with both hydrophilic and hydrophobic compounds [128]. However, in many studies involving proteins as wall materials, the focus is on investigating their interaction with other wall materials (see Table 2 and Section 4.4), particularly polysaccharides. This is because the combination of protein and polysaccharide results in heightened electrostatic interactions between the two components [129].

For instance, Fu et al. [143] observed that capsules formed with protein isolate during the encapsulation of vitamin E exhibited a collapsed spherical shape with surface cracks and roughness. This irregular morphology was attributed to the uneven dispersion of the droplet during the spray-drying process. The authors concluded that the incorporation of a polysaccharide into the protein dispersion improved homogenization, resulting in a more stable particle size and a regular morphology. In another study by Khalilvandi-Behroozyar et al. [144], the encapsulation of a polyunsaturated oil, specifically fish oil, with casein was evaluated. The findings indicated that effective oil protection was achieved when casein was used in a ratio equal to or less than the oil, such as 1:1 or 1:2 (casein–oil). Moreover, the combination of a polysaccharide with the protein enhanced encapsulation efficiency and prolonged the storage period of the encapsulated components.

### 4.3. Lipid-Based Wall Materials

Lipids are wall materials that are rarely used alone (see Section 4.4). The role of lipids in encapsulation processes is to enhance the gelling properties and viscosity of dispersions based on polysaccharides and proteins [145].

Some studies use lipid materials to create emulsions which are subsequently subjected to the spray-drying process using another wall material. For instance, Salminen et al. [146] developed two emulsions: one based on triacylglycerol with lecithin and saponins, and the other based on triacylglycerol and the compound to be encapsulated (fish oil). The authors combined both emulsions to induce crystallization and then mixed them with maltodextrin as the wall material for the drying process.

### 4.4. Spray-Drying with Wall Material Mixtures

The evaluation of different types of wall materials has been conducted during spray-drying of the same compound to compare their encapsulation efficiency, morphology, and the properties preserved in each encapsulated compound. This comparison aims to select the most suitable or best-performing wall material. Table 3 presents the combinations of polysaccharides, proteins and lipids used in spray-drying processes for the encapsulation of bioactive compounds. However, most of the research focuses on creating polymer blends to examine whether the combination of their characteristics leads to improvements in the properties of the encapsulation compounds. The following are the five most interesting findings in the encapsulation of bioactive components using material blends:

- The type and concentration of polysaccharides, lipids, and/or proteins have an impact on the encapsulation efficiency and capsule morphology [147].
- Blending materials of different nature enables the encapsulation of various type of bioactive compounds [148,149,150,151].
- Polysaccharides have the greatest influence on the yield within the wall material blends [152].
- Polysaccharides can be blended with other polysaccharides [153], lipids [154], and/or proteins [155].
- Lipids enhance the morphological characteristics of the capsules when combined with polysaccharides and/or proteins [154].

**Table 3:** Encapsulation of bioactive compounds with wall material mixtures.

Wall Material	Core Material	Concentration: Wall Material: Core Material	Conditions (Feed Rate, Inlet Air, Outlet Air)	Particles (Shape/Morphology, Particle Size Distribution)	Process Yield/Encapsulation Efficiency	Encapsulated Compounds	References
CH/MD	Tuna fish oil	15:15:10–40% *w/w*/*w*	0.7 L/h, 180 °C, 85 °C	nr./nr., nr.	nr./81–91%	Anisidine	[156]
Lentil-PI/MD	Flaxseed oil	16.5–19:1% *w/w*	3 mL/min, 135 °C, 95 °C	Spherical/Wrinkled, nr.	nr./nr.	Oil and Thiobarbituric acid	[157]
Lentil-PI/κ-Carr/MD	Flaxseed oil	16.5–19:1:5–7.5% *w/w*/*w*	3 mL/min, 135 °C, 95 °C	Spherical/Wrinkled, nr.	nr./85%	Oil and Thiobarbituric acid	[157]
Lentil-PI/ι-Carr/MD	Flaxseed oil	16.5–19:1:5–7.5% *w/w*/*w*	3 mL/min, 135 °C, 95 °C	Spherical/Wrinkled, nr.	nr./83–84%	Oil and Thiobarbituric acid	[157]
Kidney bean-PI/κ-Carr	Shrimp oil	10:0.1:0.1–1% *w/w*/*w*	5 mL/min, 180 °C, 105 °C	Spherical/Wrinkled, 2.5–6.4 μm	nr./43–89%	Fatty acids (C14, C15, C16, C17, C18, C20, C23, C24, SFA, MUFA, and PUFA)	[158]
GA/CH/Apple pectin	*Satureja khuzistanica* Jamzad extract	10:1% *w/w*	3.5 mL/min, 115 °C, nr.	Semi-spherical/Smooth, 2–5 μm	nr./58%	Phenolic compounds	[96]
GA/CH/Apple pectin	*Satureja rechingeri* Jamzad extract	10:1% *w/w*	3.5 mL/min, 115 °C, nr.	Semi-spherical/Smooth, 2–5 μm	nr./54%	Phenolic compounds	[96]
HCP/Gelatin	Turmeric oleoresin	30:1:15% *w/w*/*w*	6 mL/min, 170 °C, 80 °C	Spherical/Smooth, 2–20 μm	40%/72%	Phenolic compounds and curcumin	[152]
MD/Gelatin	Turmeric oleoresin	26:0.6:15% *w/w*/*w*	6 mL/min, 170 °C, 80 °C	Spherical/Smooth, 2–20 μm	25%/52%	Phenolic compounds and curcumin	[152]
Casein/Pectin	Grape (*Vitis labrusca*) by-product extract	12.5:12.5:1.41% *w/w*/*w*	10–14 mL/min, 120–160 °C, 68–98 °C	Spherical/Smooth, 10 μm	3–19%/60–83%	Phenolic compounds and anthocyanins	[159]
WPI/Rice-PC	Baltic herring (*Clupea harengus membras*) oil	7.5:7.5:15% *w/w*/*w*	17 kg/h, 123–129 °C, 72–78 °C	Non-spherical/Porous, 56 μm	nr./40–50%	Fatty acids (C14, C16, C18, C20, C22, C24, SFA, MUFA, and PUFA)	[160]
WPI/MD	Mix (paprika-cinnamon oleoresin)	2.5–7.5:1:1%*w/ratio/ratio*	6 mL/min, 150 °C, 80 °C	Spherical/Porous, 17–19 μm	40–43%/90–96%	Carotenoids	[80]
WP/Mo-Starch	Capsaicin	1–9:1–9:20 *ratio/ratio/%w*	nr., 185 °C, 85 °C	Spherical/Wrinkled, 1.2–51.6 μm	9–64%/50–94%	Capsaicin	[138]
WPI/MD	Gurum seed oil	2:1:1 ratio	20 mL/min, 180 °C, 80 °C	Spherical/Withered, 3–25 μm	85%/91%	Oil	[161]
GA/MD	Gurum seed oil	2:1:1 ratio	20 mL/min, 180 °C, 80 °C	Spherical/Withered, 2–10 μm	93%/97%	Oil	[161]
WPI/GA/MD	Gurum seed oil	1:1:1:1 ratio	20 mL/min, 180 °C, 80 °C	Spherical/Withered, 3–10 μm	90%/93%	Oil	[161]
MD/GA	Mamey (*Pouteria sapota*) pulp	10:5–10:nr.%/%/nr.	10 mL/min, 160 °C, 62–81 °C	nr./nr., 3 μm	nr./nr.	Carotenoids	[162]
MD/Moringa oleitera gum	Mamey (*Pouteria sapota*) pulp	10:5–10:nr.%/%/nr.	10 mL/min, 160 °C, 62–81 °C	nr./nr., 3 μm	nr./nr.	Carotenoids	[162]
MD/GA	Carriot (*Daucus carota*) pulp	10:5–10:nr.%/%/nr.	10 mL/min, 160 °C, 60–87 °C	nr./nr., 3 μm	nr./nr.	Carotenoids	[162]
MD/Moringa oleitera gum	Carriot (*Daucus carota*) pulp	10:5–10:nr.%/%/nr.	10 mL/min, 160 °C, 60–87 °C	nr./nr., 3 μm	nr./nr.	Carotenoids	[162]
CH/GA/MD	Petai leaf extract	0–1:75:25:2.5% *w/w*/*w/w*	20 mL/min, nr., 80 °C	Spherical/Collapsed, nr.	nr./nr.	Phenolic compounds	[163]
HP-βCD/MD	Grape cane extract	2.2:10:100% *w/w*/*v*	nr., 130 °C, 71 °C	Semi spherical/Smooth, 11 μm	84%/81%	Phenolic compounds (protocatechuic acid-*O*-hexoside, protocatechuic acid, ethyl protocatechuate, protocatechuic aldehyde, gallic acid, caftaric acid, ellagic acid pentoside, and hydroxybenzaldehyde), flavonoids (eriodictyol, quercetin-*O*-glucoside, quercetin-3-*O*-glucuronide, and astilbin), and stilbenes (resveratrol, stilbenoid tetramer, pallidol, ε-viniferin, stilbene, and restrytisol)	[164]
GA/WPI	Basil (*Ocimum basilicum* L.) essential oil	2:2:1% *w/w*	3 mL/min, 150 °C, nr.	Spherical/Wrinkled, 4.2 μm	71%/78%	Essential oil	[105]
WPI/MD	Basil (*Ocimum basilicum* L.) essential oil	2:2:1% *w/w*	3 mL/min, 150 °C, nr.	Spherical/Wrinkled, 3.2 μm	66%/87%	Essential oil	[105]
GA/WPI/MD	Basil (*Ocimum basilicum* L.) essential oil	1.3:1.3:1% *w/w*/*w*	3 mL/min, 150 °C, nr.	Spherical/Wrinkled, 0.6 μm	76%/83%	Essential oil	[105]
MD/Low methoxylated pectin/Sunflower wax	Flaxseed oil	3–12:1–2:1–2:1–15% *w/w*/*w/w*	4 mL/min, 135 °C, nr.	Spherical/Wrinkled, 12.9 μm	nr./44–71%	Carotenoids	[154]
MD/Mo-Starch	Fish oil	24:8:8% *w/w*/*w*	40 L/min, 190 °C, 100 °C	Spherical/Wrinkled, 0.3–69.2 μm	nr./69%	Fatty acids (saturated, monounsaturated, and polyunsaturated)	[165]
GA/MD	Vitamin A	7.5:7.5:2% *w/w*/*w*	4 mL/min, 150 °C, 80 °C	Spherical/Irregular, 0.1–0.2 μm	20%/96%	Vitamin A	[67]
Starch/GA	Vitamin A	7.5:7.5:2% *w/w*/*w*	4 mL/min, 150 °C, 80 °C	Spherical/Irregular, 0.1–0.2 μm	7%/97%	Vitamin A	[67]
Starch/MD	Vitamin A	7.5:7.5:2% *w/w*/*w*	4 mL/min, 150 °C, 80 °C	Spherical/Irregular, 0.1–0.2 μm	19%/97%	Vitamin A	[67]
Starch/GA/MD	Vitamin A	5:5:5:2% *w/w*/*w/w*	4 mL/min, 150 °C, 80 °C	Spherical/Irregular, 0.1–0.2 μm	20%/98%	Vitamin A	[67]
MD/Mo-Starch/WP	Fingered citron extract	33:33:33:10% *w/w*/*w/w*	17–21 mL/min, 185 °C, 80 °C	Spherical/Irregular, 27.5 μm	76%/71%	Phenolic compounds	[166]
GA/Mo-Starch/WP	Fingered citron extract	33:33:33:10% *w/w*/*w/w*	17–21 mL/min, 185 °C, 80 °C	Spherical/Irregular, 22.5 μm	81%/76%	Phenolic compounds	[166]
GA/MD/WP	Fingered citron extract	33:33:33:10% *w/w*/*w/w*	17–21 mL/min, 185 °C, 80 °C	Spherical/Irregular, 14.5 μm	86%/79	Phenolic compounds	[166]
GA/MD/Mo-Starch	Fingered citron extract	33:33:33:10% *w/w*/*w/w*	17–21 mL/min, 185 °C, 80 °C	Spherical/Irregular, 22.5 μm	89%/86%	Phenolic compounds	[166]
GA/MD/Mo-Starch/WP	Fingered citron extract	25:25:25:25:10% *w/w*/*w/w*/*w*	17–21 mL/min, 185 °C, 80 °C	Spherical/Irregular, 17.5 μm	78%/84%	Phenolic compounds	[166]
Ar-Starch/GA	Blackberry (*Rubus fruticosus*) pulp	0.5–2:1:1% *w/w*/*w*)	0.2 kg/h, 100–150 °C, n.r.	Spherical/Withered, 50–120 μm	29–57/nr.	Ascorbic acid and anthocyanins	[167]
MD/GA	Chipilin (*Crotalaria longirostrata*) extract	15:2:1% *w/w*/*w*	3 mL/min, 120 °C, 60 °C	Amorphous/Irregular, 3–8 μm	47%/90%	Phenolic compounds	[85]
MD/*Cajanus cajan* gum	Chipilin (*Crotalaria longirostrata*) extract	15:2:1% *w/w*/*w*	3 mL/min, 120 °C, 60 °C	Amorphous/Irregular, 3–8 μm	51%/78%	Phenolic compounds	[85]
MD/Cocoa shell pectin	Chipilin (*Crotalaria longirostrata*) extract	15:2:1% *w/w*/*w*	3 mL/min, 120 °C, 60 °C	Amorphous/Irregular, 3–8 μm	62%/75%	Phenolic compounds	[85]
MD/*Cajanus cajan* protein	Chipilin (*Crotalaria longirostrata*) extract	15:2:1% *w/w*/*w*	3 mL/min, 120 °C, 60 °C	Amorphous/Irregular, 3–8 μm	61%/93%	Phenolic compounds	[85]
MD/SPI	Chipilin (*Crotalaria longirostrata*) extract	15:2:1% *w/w*/*w*	3 mL/min, 120 °C, 60 °C	Amorphous/Irregular, 3–8 μm	62%/65%	Phenolic compounds	[85]
MD/PI	Essential avocado oil	10:5 *w/w* ratio	5 mL/min, 160 °C, 90 °C	Spherical/Aggregates, 0.1–1 μm	nr./62%	Essential oil	[148]
OSA-MD	Essential avocado oil	10:5 *w/w* ratio	5 mL/min, 160 °C, 90 °C	Spherical/Aggregates, 0.1–1 μm	nr./45%	Essential oil	[148]
OSA-MD/PI	Essential avocado oil	9:1:5 *w/w*/w ratio	5 mL/min, 160 °C, 90 °C	Spherical/Aggregates, 0.1–1 μm	nr./61%	Essential oil	[148]
λ-Carr/GA/MD	Propolis extract	1:0.2:1:1% *w/w*/*w/v*	nr., nr., nr.	Spherical/Aggregates, 0.5–6 μm	45–64%/nr.	Phenolic compounds	[168]
Ca-Starch/GA	Lemongrass (*Cymbopogon citratus*) essential oil	1–9:1:1–4 ratio	nr., 150–200 °C, nr.	Spherical/Smooth, nr.	nr./43–92%	Essential oil	[169]
MD/GA	Horseradish leaf (*Armoracia rusticana* L.) juice	20–80:20–80:20–80 ratio	0.33 L/h, 120 °C, 80 °C	nr./nr., 3.8–4.3 μm	nr./nr.	Phenolic compounds, rutin, epicatechin, catechin and sinapic acid	[77]
MD/GA	Horseradish root (*Armoracia rusticana* L.) juice	20–80:20–80:20–80 ratio	0.33 L/h, 120 °C, 80 °C	nr./nr., 3.6–3.7 μm	nr./nr.	Phenolic compounds, rutin, epicatechin, catechin and sinapic acid	[77]
MD/GA	Noni (*Morinda citrifolia* L.) juice	5–9:1:5:1% *w/w*/*w*	nr., 170 °C, 90 °C	Semi-spherical/Wrinkled, 95–106 μm	nr./nr.	Phenolic compounds	[170]
MD/WPC/GG	Rape seed oil	15.4:3.9:9.5% *w/w*/*w*	77 mL/min, 130 °C, 90 °C	Irregular/Porous, 5–75 μm	29%/90%	Fatty acids (C14, C16, C18, SFA, MUFA, and PUFA)	[155]
MD/WPC/GG	Flax seed oil	15.4:3.9:9.5% *w/w*/*w*	77 mL/min, 130 °C, 90 °C	Irregular/Porous, 5–75 μm	30%/88%	Fatty acids (C14, C16, C18, SFA, MUFA, and PUFA)	[155]
MD/WPC/GG	Safflower seed oil	15.4:3.9:9.5% *w/w*/*w*	77 mL/min, 130 °C, 90 °C	Irregular/Porous, 5–75 μm	30%/82%	Fatty acids (C14, C16, C18, SFA, MUFA, and PUFA)	[155]
Gelatin/Chia mucilage	Oregano (*Origanum vulgare*) essential oil	1:1:1% *w/w*/*w*	nr., 160–180 °C, nr.	Spherical/Aggregates, 38–120 μm	81–89%/85–96%	Essential oil	[171]
Gelatin/GA	Oregano (*Origanum vulgare*) essential oil	1:1:1% *w/w*/*w*	nr., 160–180 °C, nr.	Spherical/Aggregates, 18–85 μm	72–88%/89–95%	Essential oil	[171]
Casein/MD	Thyme (*Thymus* *vulgaris*) essential oil	4.17:80:20% *w/w*/*w*	7–5 mL/min, 110 °C, 70 °C	Spherical/Irregular, 0.87 μm	nr./89%	Phenolic compounds	[172]
OSA-Starch/MD	β-carotene	1:1–3:1 ratio	1100 mL/h, 185 °C, nr.	Spherical/Wrinkled, 2–6 μm	nr.	β-carotene	[173]
CH/WPI	Garlic bulbs extract	1:1:nr.% *w/w*/*nr*.	nr., 160 °C, nr.	Spherical/Aggregates, 2–10 μm	nr./nr.	Phenolic compounds	[129]
Zein/NaCas	Curcumin	10:10% *w/w*	120 L/min, 100 °C, nr.	Spherical/Irregular, 143 nm	nr./90–95%	Curcumin	[149]
κ-Carr/MP	Tuna oil	1:1–50:1 ratio	2.5 mL/min, 180 °C, 80 °C	Spherical/Smooth, 3–6 μm	82–97%/91–97%	Oil	[174]
λ-Carr/MP	Tuna oil	1:1–50:1 ratio	2.5 mL/min, 180 °C, 80 °C	Spherical/Smooth, 3–5 μm	82–99%/91–98%	Oil	[174]
MD/Gelatin	Turmeric (*Curcuma longa* L.) oleoresin	12–26:0.6–6:15% *w/w*/*w*	1.4–8.6 mL/min, 124–190 °C, nr.	Spherical/Aggregates, nr.	nr./4–77%	Phenolic compounds	[175]
Gelatin/Sodium hexametaphosphate	Anchovy oil	8:0.5:30% *w/w*/*w*	nr., 160 °C, 94 °C	Oval/Rough, 40–60 μm	96%/100%	Oil	[176]
GA/MD	Fish oil	15:15:15% *w/w*/*w*	nr., 118–120 °C, nr.	Spherical/Rugged, 13–105 μm	nr./51–57%	Oil	[177]
Casein/Pectin/MD	Fish oil	15:15:15:15% *w/w*/*w/w*	nr., 118–120 °C, nr.	Spherical/Rugged, 11–68 μm	nr./65–68%	Oil	[177]
WPC/Hawthorn pectin	Grape seed oil	1–1.5:1–1.5:1% *w/w*/*w*	40 mL/min, 170 °C, 85 °C	Spherical/Smooth, 1.6–2.6 μm	nr./65–71%	Oil	[178]
Ar-Starch/GA	Blackberry (*Rubus fruticosus*) pulp	15.4:10.2% *w/w*	0.2 kg/h, 100–150 °C, nr.	Spherical/Aggregates, 50.9–119.8 μm	29–57%/nr.	Phenolic compounds	[179]
MD/SP	Lemon by-product aqueous extract	5:1 ratio	4 mL/min, 125 °C, 55 °C	Spherical/Smooth, nr.	58–67%/nr.	Phenolic compounds and flavonoids	[89]
MD/ι-Carr	Lemon by-product aqueous extract	9:1 ratio	4 mL/min, 125 °C, 55 °C	Spherical/Smooth, nr.	56–59%/nr.	Phenolic compounds and flavonoids	[89]
Gelatin/MD	Fish oil	7.5:32.5:10% *w/w*	nr., 180 °C, 80 °C	Spherical/Smooth, nr.	nr./85%	Oil	[180]
κ-Carr/MD	Fish oil	1:38.5:10% *w/w*	nr., 180 °C, 80 °C	Spherical/Smooth, nr.	nr./67%	Oil	[180]
Gelatin/κ-Carr	Fish oil	7.5:31.5:10% *w/w*	nr., 180 °C, 80 °C	Spherical/Smooth, nr.	nr./75%	Oil	[180]
SC/βCD	Kenaf (*Hibiscus cannabinus* L.) seed oil	2:1:1 ratio	8 g/min, 160 °C, nr.	Spherical/Smooth holes, 37.3 μm	nr./93%	Oil	[181]
GA/βCD	Kenaf (*Hibiscus cannabinus* L.) seed oil	2:1:1 ratio	8 g/min, 160 °C, nr.	Spherical/Smooth holes, 30.6 μm	nr./95%	Oil	[181]
GA/SC/βCD	Kenaf (*Hibiscus cannabinus* L.) seed oil	4:1:1:1 ratio	8 g/min, 160 °C, nr.	Spherical/Smooth holes, 25.4 μm	nr./90%	Oil	[181]
Brea gum/Inulin	Corn oil	20:10–20:10% *w/w*/*w*	nr., 150 °C, 60 °C	Semi-spherical/Dents, 0.8–18 μm	nr./74–92%	Oil	[111]
GA/Inulin	Corn oil	20:10–20:10% *w/w*/*w*	nr., 150 °C, 60 °C	Semi-spherical/Dents, 15 μm	nr./87–89%	Oil	[111]
MD/Carr	*Pouzolzia zeylanica* extract	5–15:0.06–0.1:nr.% *w/w*/*nr*.	nr., 180 °C, nr.	nr./nr., 6 μm	nr./nr.	Phenolic compounds, anthocyanins, flavonoids, and tannins	[182]
GA/MD	Grape seed oil	15:15:10% *w/w*/*w*	350 mL/h, 180 °C, 105 °C	Spherical/Collapsed, 27 μm	nr./63%	Fatty acids (C14, C16, C18, C20, SFA, MUFA, and PUFA) and phenolic compounds	[110]
MD/GA	Algal (*Tetraselmis chuii*) biomass	60:40:1 *ratio/ratio/%w*	2.5 mL/min, 150 °C, 60 °C	Spherical/Rough, 3.5–13.7 μm	22–45%/54–84%	Phenolic compounds, β-carotene, and carotenoids	[99]
MD/GA	Peach palm peel extract	7.6:7.6:nr.% *w/w*/*nr*.	12.6 mL/min, 160 °C, 70 °C	Irregular/Irregular, nr.	72%/67%	β-carotene	[183]
WPI/SC	Conjugated linoleic acid	1:4:8% *w/w*/*w*	nr., 160 °C, 80 °C	Spherical/Irregular, 10–25 μm	nr./96%	Conjugated linoleic acid	[141]
SOS-Starch/MD	Chili seed oil	1–5:1:20–45% *w/w*/*w*	nr., 160 °C, 80 °C	Polyhedral/Irregular, 3–20 μm	nr.	Fatty acids (C14, C16, C18, C20, C22, SFA, MUFA, PUFA, and UFA)	[151]
R-Starch/Mo-Starch/MD/HP	Orange essential oil	0–30:0–30:0–30:0–30:15% *w/w*/*w/w*/*w*	3.75 mL/min, 180 °C, 85 °C	Spherical/Rough, 30–40 μm	38–82%/45–96%	D-Limonene	[70]
R-Starch/Mo-Starch	Orange essential oil	0–30:15% *w/w*/*w*	3.75 mL/min, 180 °C, 85 °C	Spherical/Rough, 30–40 μm	65–73%/96–99%	D-Limonene	[70]
Mo-Starch/MD	Orange essential oil	0–30:15% *w/w*/*w*	3.75 mL/min, 180 °C, 85 °C	Spherical/Rough, 30–40 μm	58%/99%	D-Limonene	[70]
MD/HP	Orange essential oil	0–30:15% *w/w*/*w*	3.75 mL/min, 180 °C, 85 °C	Spherical/Rough, 30–40 μm	33%/44%	D-Limonene	[70]
R-Starch/HP	Orange essential oil	0–30:15% *w/w*/*w*	3.75 mL/min, 180 °C, 85 °C	Spherical/Rough, 30–40 μm	87%/58%	D-Limonene	[70]
MD/GA	Drumstick (*Moringa oleifera*) oil	25–75:25–75:30% *w/w*/*w*	10 g/min, 180 °C, 85 °C	Spherical/Smooth, 23–28 μm	nr./83–91	Oil	[184]
MD/WPC	Drumstick (*Moringa oleifera*) oil	25–75:25–75:30% *w/w*/*w*	10 g/min, 180 °C, 85 °C	Spherical/Smooth, 11–18 μm	nr./66–73%	Oil	[184]
MD/GA	Spent coffee ground extract	1:1:10 *w/w*/*v* ratio	108 mL/min, 100 °C, nr.	Spherical/Withered, <30 μm	nr./25–80%	Phenolic compounds and flavonoids	[94]
MD/Moringa oleitera gum	Tender coconut (*Cocos nucifera*) water	10–50:0.5–1.5% *w/w*	0.4 kg/h, 100–140 °C, 90–97 °C	Spherical/Irregular, 2.5–15 μm	9–38%/38–95%	Phenolic compounds	[185]
MD-CAP	Vitamin A	70:30:1% *w/w*/*w*	2 mL/min, 120 °C, 74 °C	Semi-spherical/Dented, 2–4 μm	80–81%/48–100%	Vitamin A	[186]
MD-SC	Vitamin E	70:30:1% *w/w*/*w*	2 mL/min, 120 °C, 74 °C	Semi-spherical/Dented, 2–4 μm	77–85%/23–29%	Vitamin E	[186]
MD/CAP	Vitamin A	2.2–6.6:2.2–6.6:6% *w/w*/*w*	1–5 mL/min, 110–130 °C, 55–60 °C	Spherical/Irregular, 3–15 μm	nr./59–63%	Vitamin A	[150]
MD/GV	Propolis	30:0.3:0.123% *w/w*/*w*	8 mL/min, 120 °C, 70–74 °C	Deformed spherical/Smooth, nr.	60%/81–89%	Phenolic compounds	[91]
MD/GA	Propolis	30:0.3:0.123% *w/w*/*w*	8 mL/min, 120 °C, 70–74 °C	Deformed spherical/Smooth, nr.	68%/84–93%	Phenolic compounds	[91]
C-Zein/-βCD	α-Tocopherol	2.5:1.85:0.5% *w/w*/*w*	7–9 mL/min, 110–180 °C, nr.	Spherical/Smooth holes, 10 μm	44–77%/31–42%	α-Tocopherol	[137]
Gelatin/GA	Fish oil	0.5:0.5:2% *w/w*/*w*	6 mL/min, 190 °C, 90 °C	Spherical/Smooth, 2–6 μm	nr./87–94%	Oil	[187]
WPC/GA	Pumpkin (*Cucurbita* spp.) seed oil	5:5:5% *w/w*/*w*	0.8 L/h, 160 °C, 60 °C	Spherical/Cracked, nr.	65%/60%	Oil	[153]
WPC/C-Starch	Pumpkin (*Cucurbita* spp.) seed oil	5:5:5% *w/w*/*w*	0.8 L/h, 160 °C, 60 °C	Spherical/Cracked, nr.	60%/30%	Oil	[153]
WPC/Ma-Starch	Pumpkin (*Cucurbita* spp.) seed oil	5:5:5% *w/w*/*w*	0.8 L/h, 160 °C, 60 °C	Spherical/Cracked, nr.	55%/40%	Oil	[153]
WPC/MD	Pumpkin (*Cucurbita* spp.) seed oil	5:5:5% *w/w*/*w*	0.8 L/h, 160 °C, 60 °C	Spherical/Cracked, nr.	56%/93%	Oil	[153]
WPC/Glucose	Pumpkin (*Cucurbita* spp.) seed oil	5:5:5% *w/w*/*w*	0.8 L/h, 160 °C, 60 °C	Spherical/Cracked, nr.	56%/95%	Oil	[153]
WPC/Sucrose	Pumpkin (*Cucurbita* spp.) seed oil	5:5:5% *w/w*/*w*	0.8 L/h, 160 °C, 60 °C	Spherical/Cracked, nr.	53%/96%	Oil	[153]
WPC/Lactose	Pumpkin (*Cucurbita* spp.) seed oil	5:5:5% *w/w*/*w*	0.8 L/h, 160 °C, 60 °C	Spherical/Cracked, nr.	48%/95%	Oil	[153]
WPC/Maltose	Pumpkin (*Cucurbita* spp.) seed oil	5:5:5% *w/w*/*w*	0.8 L/h, 160 °C, 60 °C	Spherical/Cracked, nr.	56%/95%	Oil	[153]
Pectin/WPC	Folic acid	0.1–2:0.1–0.3% *w/w*/*w*	450 mL/h, 180 °C, 90 °C	Spherical/Smooth, 2–10 μm	nr./nr.	Folic acid	[188]

nr.: not reported; Ar: arrowroot; C: corn; Ca: cassava; Carr: carrageenan; CAP: capsul; CH: chitosan; βCD: β-cyclodextrin; GA: gum Arabic; GG: gum guar; GV: gum vinal; C14: myristic acid; C15: pentadecanoic acid; C16: palmitic acid; C17: heptadecanoic acid; C18: stearic acid; C20: cetoleic acid; C22: adrenic acid; C23: tricosanoic acid; C24: tetracosanoic acid; HCP: Hi-cap100; HP-βCD: hydroxypropyl β-cyclodextrin; HP: hydrolyzed protein; Ma: manioc; MD: maltodextrin; Mo: modified; MP: myofibrillar protein; MUFA: monounsaturated fatty acid; NaCas: sodium caseinate; OSA: octenyl succinic anhydride; PC: protein concentrate; PI: protein isolate; PUFA: polyunsaturated fatty acid; R: rice; SC: sodium caseinate; SFA: saturated fatty acid; SOS: sodium octenylsuccinate; SP: soybean protein; SPI: soy protein isolate; UFA: unsaturated fatty acid; WP: whey protein; WPC: whey protein concentrate; WPI: whey protein isolate.

## 5. Concluding Remarks

Spray-drying is a versatile technique that finds wide application in various industries, such as food, pharmaceutical, and cosmetic. Its adaptability to meet industry-specific requirements makes it a popular choice for encapsulating bioactive compounds. The selection of an appropriate wall material plays a crucial role in spray-drying processes, as it determines the formation of capsules, their stability, and the degree of protection provided to the core material (the desired compounds). Polysaccharides, due to their cost-effectiveness and favorable properties, are the most commonly used materials in encapsulation. They can be combined due to their cost and properties, which allow them to be mixed with other polysaccharides, proteins, or lipids to enhance encapsulation yield, efficiency, morphology, and stability. By carefully selecting one or more wall materials, the operating conditions of spray-drying, such as air temperature, airflow rate, and feed rate, can be manipulated to achieve optimal yields, encapsulation efficiency, and the desired physical and physicochemical properties in the encapsulated materials, while preserving their bioactivity.

## Figures and Tables

**Table 1:** Encapsulation of bioactive compounds with polysaccharide-based wall materials.

Wall Material	Core Material	Concentration (Wall Material: Core Material)	Conditions (Feed Rate, Inlet Air, Outlet Air)	Particles (Shape/Morphology, Particle Size Distribution)	Process Yield/Encapsulation Efficiency	Encapsulated Compounds	References
A-Starch	*Hibiscus sabdariffa* extract	10–15:nr.% *w*/*nr*.	4 mL/min, 100–140 °C, 56–84 °C	Ovoid/Collapsed, 10 μm	nr./15–69%	Antimicrobial compounds	[63]
C-Starch	Corn oil	2:1% *w/w*	nr., 180 °C, 115 °C	Semispherical/Shrunken, 12–38 μm	64%/89%	Oil	[64]
M-Starch	Pumpkin oil	15.4:10.2% *w/w*	77 mL/min, 130 °C, 90 °C	Spherical/Withered, 51.6–77.3 μm	nr./42%	α-Tocopherol, γ-tocopherol, squalene, spinasterol, β-sitosterol, stigmastatrienol, stigmasterol, and stigmastadienol	[65]
M-Starch	Ascorbyl palmitate	3:1 ratio	6.3 mL/min, 185 °C, 70 °C	nr./nr., nr.	nr.	Ascorbyl palmitate	[66]
Mo-Starch	Vitamin A	15:2% *w/w*	4 mL/min, 150 °C, 80 °C	Spherical/Irregular, 0.1–0.2 μm	17%/89%	Vitamin A	[67]
Mo-Starch	Vitamin B1	1:0.125% *w/w*	4 mL/min, 120 °C, 50–67 °C	Spherical/Irregular, 0.1–3.6 μm	25%/95%	Vitamin B1	[68]
Mo-Starch	Fish oil	15:nr.% *w/nr*.	0.7 L/h, 180 °C, nr.	Spherical/Smooth holes, 10–26 μm	nr./76%	Oil	[69]
Mo-Starch	Orange essential oil	30:15% *w/w*	3.75 mL/min, 180 °C, 85 °C	Spherical/Rough, 30–40 μm	73%/99%	D-Limonene	[70]
P-Starch	Gallic acid	20:0.1–10% *w/w*	nr., 160 °C, 75 °C	Spherical/Withered, 1–15 μm	nr./70–84%	Gallic acid	[71]
R-Starch	Pumpkin oil	15.4:10.2% *w/w*	77 mL/min, 130 °C, 90 °C	Spherical/Withered, 41.1–67.7 μm	nr./35%	α-Tocopherol, γ-tocopherol, squalene, spinasterol, β-sitosterol, stigmastatrienol, stigmasterol, and stigmastadienol	[65]
R-Starch	Vanilla extract	10–15:nr.% *w/nr*.	4 mL/min, 100–140 °C, 70–94 °C	Polyhedral/Irregular, 2–7 μm	20–44%/15–69%	Vanillin	[72]
R-Starch	Orange essential oil	30:15% *w/w*	3.75 mL/min, 180 °C, 85 °C	Spherical/Rough, 30–40 μm	73%/37%	D-Limonene	[70]
SOS-Starch	*Nigella sativa* seeds oil	18.75:6.25:1% *w/w*/*w*	nr., 140 °C, 95–98 °C	Spherical/Irregular, 1–30 μm	nr./80–90%	Volatile compounds	[73]
T-Starch	Almond oil	20:10–20% *w/w*	10.6 g/min, 145 °C, nr.	Spherical/Aggregates, 1.6–31.1 μm	56%/38–45%	Peroxides	[74]
T-Starch	L-ascorbic acid	30:10% *w/w*	19.5 g/min, 145 °C, 80 °C	Spherical/Aggregates, 2–10 μm	nr.	L-ascorbic acid	[75]
W-Starch	Pumpkin oil	15.4:10.2% *w/w*	77 mL/min, 130 °C, 90 °C	Spherical/Withered, 41.4–70.9 μm	nr./68%	α-Tocopherol, γ-tocopherol, squalene, spinasterol, β-sitosterol, stigmastatrienol, stigmasterol, and stigmastadienol	[65]
Starch	Tea (*Camelia sinensis* L.) leaves extract	1.5:1% *w/w*	4 mL/min, 115 °C, 65 °C	Ovoid/Smooth, 10–50 μm	59/nr.	Phenolic compounds	[76]
Starch	Horseradish leaf (*Armoracia rusticana* L.) juice	20–80:20–80 ratio	0.33 L/h, 120 °C, 80 °C	nr./nr., 9.5–14.1 μm	nr./nr.	Phenolic compounds, rutin, epicatechin, catechin and sinapic acid	[77]
HCP	Borage seed oil	10–30:13% *w/w*	10 mL/min, 170 °C, 80 °C	Asymmetrical/Dented, 4–15 μm	nr./89%	Oil	[78]
HCP	Borage seed oil/Curcumin	10–30:13:0.6% *w/w*/*w*	10 mL/min, 170 °C, 80 °C	Asymmetrical/Dented, 4–15 μm	nr./91%	Curcumin	[78]
HCP	Borage seed oil/Resveratrol	10–30:13:0.4% *w/w*/*w*	10 mL/min, 170 °C, 80 °C	Asymmetrical/Dented, 4–15 μm	nr./88%	Resveratrol	[78]
HCP	Borage seed oil/Curcumin/Resveratrol	10–30:13:0.6:0.4% *w/w*/*w/w*	10 mL/min, 170 °C, 80 °C	Asymmetrical/Dented, 4–15 μm	nr./93%	Curcumin and resveratrol	[78]
C-MD	Corn mint (*Mentha arvensis* L.) essential oil	20–30:0.5–2% *w/w*	4–10 mL/min, 130–150 °C, nr.	nr./nr., nr.	69%/99%	Menthol, menthone, menthyl acetate, isomenthone, caryphyllene, eucalyptol, α-terpineol, δ-cadinene, neoisomenthol, pulegone, β-bourbonene, and nerolidol	[79]
MD	Mix (paprika-cinnamon oleoresin)	10:1:1%w/ratio/ratio	6 mL/min, 150 °C, 80 °C	Spherical/Porous, 33 μm	34%/65%	Carotenoids	[80]
MD	Tucuma coproduct (*Astrocaryum vulgare* Mart.) almonds extract	5:5% *w/w*	7.5 mL/min, 100 °C, nr.	Spherical/Wrinkled, 2–8 μm	nr./81–96%	Phenolic compounds, flavonoids, and tannins	[81]
MD	Pumpkin oil	15.4:10.2% *w/w*	77 mL/min, 130 °C, 90 °C	Spherical/Withered, 58.0–103.0 μm	nr./70%	α-Tocopherol, γ-tocopherol, squalene, spinasterol, β-sitosterol, stigmastatrienol, stigmasterol, and stigmastadienol	[65]
MD	Red dragón fruit (*Hylocereus polyrhizus*) juice	5–15:nr.% *w/nr*.	500 mL/h, 170 °C, 70 °C	nr./nr., nr.	53–74%/nr.	Phenolic compounds	[82]
MD	Cornsilk extract	10:90% *w/w*	nr., nr., nr.	Spherical/Irregular, nr.	76–87%/99%	Phenolic compounds (gallic acid, protocateuic acid, protocatechuic aldehyde, catechin, caffeic acid, vanillin, *p*-cumaric acid, ferulic acid, 4-hydroxy benzoic acid, salicylic acid, and ellagic acid) and flavonoids	[83]
MD	Brown seaweed (*Saccharina japonica*) extract	10:100% *w/v*	30 mL/min, 230 °C, 105 °C	Spherical/Wrinkled, 9.3 μm	nr./33%	Phenolic acids (gallic acid, chlorogenic acid, gentisic acid, protocatechuic acid, p-hydroxybenzoic acid, vanillic acid, caffeic acid, and syringic acid)	[84]
MD	Vitamin B1	1:0.125% *w/w*	4 mL/min, 120 °C, 50–67 °C	Spherical/Irregular, 0.1 μm	50%/65%	Vitamin B1	[68]
MD	Vitamin A	15:2% *w/w*	4 mL/min, 150 °C, 80 °C	Spherical/Irregular, 0.1–0.2 μm	39%/95%	Vitamin A	[67]
MD	Chipilin (*Crotalaria longirostrata*) extract	17:1% *w/w*	3 mL/min, 120 °C, 60 °C	Amorphous/Irregular, 3–8 μm	64%/86%	Phenolic compounds	[85]
MD	Stevia extract	2:1 *w/w* ratio	nr., 130 °C, 87 °C	Spherical/Aggregates, <20 μm	nr./77–88%	Steviol glycoside, stevioside, rebaudioside A, rebaudioside C, and phenolic compounds	[86]
MD	Pineapple (*Ananas comosus*) peel extract	5:nr.% *w/nr*.	3.7 mL/min, 150–190 °C, 80 °C	Spherical/Aggregates, 2–13 μm	nr./nr.	Phenolic compounds	[87]
MD	Horseradish root (*Armoracia rusticana* L.) juice	20–80:20–80 ratio	0.33 L/h, 120 °C, 80 °C	nr./nr., 3.7–4.0 μm	nr./nr.	Phenolic compounds, rutin, epicatechin, catechin, and sinapic acid	[77]
MD	Chokeberry extract	2:10 *w/v* ratio	nr., nr., nr.	Spherical/Aggregates, 4–10 μm	nr./97%	Phenolic compounds, anthocyanins, and cyanidin-3-glucoside	[88]
MD	Lemon by-product aqueous extract	30–35:30% *w/v*	4 mL/min, 125 °C, 55 °C	Spherical/Smooth, nr.	56–58%/nr.	Phenolic compounds and flavonoids	[89]
MD	Greek saffron extract	5–20:1 *w/w* ratio	nr., 100 °C, nr.	Spherical/Aggregates, 2–5 μm	71–87%/55–80%	Crocins and picrocrocin	[90]
MD	Orange essential oil	30:15% *w/w*	3.75 mL/min, 180 °C, 85 °C	Spherical/Rough, 30–40 μm	29%/72%	D-Limonene	[70]
MD	Propolis	30:0.123% *w/w*/*w*	8 mL/min, 120 °C, 70–74 °C	Deformed spherical/Smooth, nr.	68%/86–98%	Phenolic compounds	[91]
MD	Fish oil	34.2:24% *w/w*	25 mL/min, 140 °C, 70–95 °C	Spherical/Wrinkled, 1 μm	nr./74–90%	Oil	[92]
MD	Blueberry (*Vaccinium corymbosum*) juice	30:70% *w/w*	7 mL/min, 180 °C, 70 °C	Spherical/Irregular, nr.	nr./nr.	Resveratrol and quercetin 3-D-galactoside	[93]
MD	Spent coffee ground extract	2:10 *w/v* ratio	108 mL/min, 100 °C, nr.	Spherical/Withered, <30 μm	nr./50–85%	Phenolic compounds and flavonoids	[94]
Mo-CH	Vitamin B1	1:0.125% *w/w*	4 mL/min, 120 °C, 50–67 °C	Spherical/Irregular, 0.6 μm	42%/95%	Vitamin B1	[68]
CH	Insulin	1:0.2% *w/w*	3 mL/min, 120 °C, 50–55 °C	Spherical/Irregular, <1.2 μm	nr./nr.	Insulin	[95]
CH	*Satureja khuzistanica* Jamzad extract	10:1% *w/w*	3.5 mL/min, 115 °C, nr.	Spherical/Smooth, 2 μm	nr./59%	Phenolic compounds	[96]
CH	*Satureja rechingeri* Jamzad extract	10:1% *w/w*	3.5 mL/min, 115 °C, nr.	Spherical/Smooth, 2 μm	nr./43%	Phenolic compounds	[96]
CH	Curcumin	0.25–0.5:1.7% *w/w*	4 mL/min, 180 °C, nr.	Spherical/Irregular, 1–5 μm	92–99%/51–72%	Curcumin	[97]
CH	Vitamin B1	1:0.125% *w/w*	4 mL/min, 120 °C, 50–67 °C	Spherical/Irregular, 0.1–0.8 μm	35%/95%	Vitamin B1	[68]
CH	Iron gluconate	3:1 ratio	nr., 160 °C, nr.	Spherical/Wrinkled, 1–10 μm	28–43%/24–38%	Iron gluconate	[98]
CH	Algal (*Tetraselmis chuii*) biomass	3:1% *w/w*	2.5 mL/min, 150 °C, 60 °C	Spherical/Rough, 1–15.2 μm	22–45%/53–73%	Phenolic compounds, β-carotene, and carotenoids	[99]
CH	Potassium phosphate	25:5–50% *w/w*	1.4–3.6 mL/min, 120–150 °C, nr.	Semi-spherical/collapsed, 1–2 μm	nr./88–92%	Potassium phosphate	[100]
CH	Cacao hull waste extract	0.4:0.1–0.9% *w/w*	2.5 cm^3^/min, 170 °C, 75–80 °C	Semi-spherical/Dented, 156–400 nm	nr./19–88%	Phenolic compounds and flavonoids	[101]
CH	Venlafaxine hydrochloride	0.5:30% *w/w*	32 m^3^/h, 160 °C, nr.	Spherical/Irregular, 3–10 μm	44–74%/37–94%	Venlafaxine hydrochloride	[102]
CH	Squalene	1:0.3–1 ratio	nr., 160 °C, 90 °C	Spherical/Smooth, 6.8 μm	nr./26%	Squalene	[103]
GA	*Satureja khuzistanica* Jamzad extract	10:1% *w/w*	3.5 mL/min, 115 °C, nr.	Semi-cubes/Rough, 2.6 μm	nr./50%	Phenolic compounds	[96]
GA	*Satureja rechingeri* Jamzad extract	10:1% *w/w*	3.5 mL/min, 115 °C, nr.	Semi-cubes/Rough, 2.6 μm	nr./38%	Phenolic compounds	[96]
GA	Guaraná (*Paullinia cupana*) peel extract	20:20–33% *w/w*	10 mL/min, 140 °C, nr.	Spherical/Irregular, 10–16 μm	nr./82–100%	Carotenoids	[104]
GA	Red dragón fruit (*Hylocereus polyrhizus*) juice	5–15:nr.% *w/nr*.	500 mL/h, 170 °C, 70 °C	nr./nr., nr.	80–91%/nr.	Phenolic compounds	[82]
GA	Brown seaweed (*Saccharina japonica*) extract	10:100% *w/v*	30 mL/min, 230 °C, 105 °C	Spherical/Wrinkled, 34.4 μm	nr./39%	Phenolic acids (gallic acid, chlorogenic acid, gentisic acid, protocatechuic acid, p-hydroxybenzoic acid, vanillic acid, caffeic acid, and syringic acid)	[84]
GA	Basil (*Ocimum basilicum* L.) essential oil	4:1% *w/w*	3 mL/min, 150 °C, nr.	Spherical/Wrinkled, 0.5 μm	66%/82%	Essential oil	[105]
GA	Vitamin B1	1:0.125% *w/w*	4 mL/min, 120 °C, 50–67 °C	Spherical/Irregular, 0.1–0.6 μm	38%/95%	Vitamin B1	[68]
GA	Vitamin A	15:2% *w/w*	4 mL/min, 150 °C, 80 °C	Spherical/Irregular, 0.1–0.2 μm	35%/88%	Vitamin A	[67]
GA	Pineapple (*Ananas comosus*) peel extract	5:nr.% *w/nr*.	3.7 mL/min, 150–190 °C, 80 °C	Spherical/Aggregates, 4–8 μm	nr./nr.	Phenolic compounds	[87]
GA	Non-dewaxed propolis extract	4:1 *w/w* ratio	8 mL/min, 120 °C, 68 °C	Spherical/Withered, 0.6 μm	nr./46%	Bioflavonoids, pinocembrin, galangin, chrysin and phenolic compounds	[106]
GA	L-ascorbic acid	1–4:1 ratio	2–7 mL/min, 140 °C, 86 °C	Spherical/Aggregates, 3–10 μm	67–83%/82–98%	L-ascorbic acid	[107]
GA	Tomato (*Solanum lycopersicum* L.) pomace extract	9.4–30.6:15% *w/w*	3.7 mL/min, 110–200 °C, nr.	Spherical/Smooth holes, 0.7–27 μm	15–44%/3–21%	Lycopene	[108]
GA	Non-dewaxed propolis extract	4:1 *w/w* ratio	0.5 mL/min, 80 °C, 50 °C	Spherical/Withered, 0.5 μm	nr./21%	Bioflavonoids, pinocembrin, galangin, chrysin and phenolic compounds	[106]
GA	Curcumin	10–20:0.1% *w/w*	4 mL/min, 150 °C, 88 °C	Spherical/Rough, 0.2–0.3 μm	29–42%/nr.	Curcumin	[109]
GA	Grape seed oil	30:10% *w/w*	350 mL/h, 180 °C, 105 °C	Spherical/Collapsed, 27 μm	nr./68%	Fatty acids (C14, C16, C18, C20, SFA, MUFA, and PUFA) and phenolic compounds	[110]
GA	Corn oil	20:20% *w/w*	nr., 150 °C, 60 °C	Semi-spherical/Dents, 0.8–10 μm	nr./89%	Oil	[111]
GA	Fish oil	15:nr.% *w/nr*.	0.7 L/h, 180 °C, nr.	Spherical/Smooth holes, 9–22 μm	nr./60%	Oil	[69]
GA	Spent coffee ground extract	2:10 *w/v* ratio	108 mL/min, 100 °C, nr.	Spherical/Withered, <30 μm	nr./35–80%	Phenolic compounds and flavonoids	[94]
SA	Tea (*Camelia sinensis* L.) leaves extract	1.5:1% *w/w*	4 mL/min, 115 °C, 65 °C	Spherical/Dented, 1–5 μm	55/nr.	Phenolic compounds	[76]
SA	Vitamin B1	1:0.125% *w/w*	4 mL/min, 120 °C, 50–67 °C	Spherical/Irregular, 0.6–1.0 μm	42%/95%	Vitamin B1	[68]
SA	L-ascorbic acid	1–4:1 ratio	2–7 mL/min, 140 °C, 86 °C	Spherical/Aggregates, 5–14 μm	35–76%/92–99%	L-ascorbic acid	[107]
SA	Olive (*Olea europaea* L.) leaves extract	0.35–2.15:1 ratio	3 mL/min, 135–195 °C, 70–90 °C	Spherical/Smooth holes, 0.25–20 μm	35–57%/54–69%	Oleuropein	[112]
SC	Brown seaweed (*Saccharina japonica*) extract	10:100% *w/v*	30 mL/min, 230 °C, 105 °C	Spherical/Wrinkled, 13 μm	nr./63%	Phenolic acids (gallic acid, chlorogenic acid, gentisic acid, protocatechuic acid, p-hydroxybenzoic acid, vanillic acid, caffeic acid, and syringic acid)	[84]
λ-Carr	Vitamin B1	1:0.125% *w/w*	4 mL/min, 120 °C, 50–67 °C	Spherical/Irregular, 0.5–1.2 μm	32%/95%	Vitamin B1	[68]
Carr	Tea (*Camelia sinensis* L.) leaves extract	1.5:1% *w/w*	4 mL/min, 115 °C, 65 °C	Spherical/Rough, 0.5–4 μm	46/nr.	Phenolic compounds	[76]
Inulin	Red dragón fruit (*Hylocereus polyrhizus*) juice	5–15:nr.% *w/nr*.	500 mL/h, 170 °C, 70 °C	nr./nr., nr.	32–46%/nr.	Phenolic compounds	[82]
Inulin	Pineapple (*Ananas comosus*) peel extract	5:nr.% *w/nr*.	3.7 mL/min, 150–190 °C, 80 °C	Spherical/Aggregates, 1–18 μm	nr./nr.	Phenolic compounds	[87]
Inulin	Tomato (*Solanum lycopersicum* L.) pomace extract	9.4–30.6:15% *w/w*	3.7 mL/min, 110–200 °C, nr.	Spherical/Smooth, 0.6–22 μm	35–50%/7–25%	Lycopene	[108]
Inulin	Blueberry (*Vaccinium corymbosum*) juice	30:70% *w/w*	7 mL/min, 180 °C, 70 °C	Spherical/Irregular, nr.	nr./nr.	Resveratrol and quercetin 3-D-galactoside	[93]
Inulin	Olive (*O. europaea* L.) leaves extract	0.34–2.15:1 ratio	nr., 135–184 °C, nr.	nr./nr., nr.	64%/87%	Phenolic compounds	[113]
Lactose	Pumpkin oil	15.4:10.2% *w/w*	77 mL/min, 130 °C, 90 °C	Spherical/Withered, 58.3–104.0 μm	nr./71%	α-Tocopherol, γ-tocopherol, squalene, spinasterol, β-sitosterol, stigmastatrienol, stigmasterol, and stigmastadienol	[65]
Lactose	Brown seaweed (*Saccharina japonica*) extract	10:100% *w/v*	30 mL/min, 230 °C, 105 °C	Spherical/Wrinkled, 127 μm	nr./30%	Phenolic acids (gallic acid, chlorogenic acid, gentisic acid, protocatechuic acid, p-hydroxybenzoic acid, vanillic acid, caffeic acid, and syringic acid)	[84]
Trehalose	Pumpkin oil	15.4:10.2% *w/w*	77 mL/min, 130 °C, 90 °C	Spherical/Withered, 85.2–176.0 μm	nr./59%	α-Tocopherol, γ-tocopherol, squalene, spinasterol, β-sitosterol, stigmastatrienol, stigmasterol, and stigmastadienol	[65]
Dextrin	Brown seaweed (*Saccharina japonica*) extract	10:100% *w/v*	30 mL/min, 230 °C, 105 °C	Spherical/Wrinkled, 43.4 μm	nr./1%	Phenolic acids (gallic acid, chlorogenic acid, gentisic acid, protocatechuic acid, p-hydroxybenzoic acid, vanillic acid, caffeic acid, and syringic acid)	[84]
Xanthan	Vitamin B1	1:0.125% *w/w*	4 mL/min, 120 °C, 50–67 °C	Spherical/Irregular, 0.1–0.7 μm	17%/95%	Vitamin B1	[68]
Skimmed milk	Chokeberry extract	2:10 *w/v* ratio	nr., nr., nr.	Spherical/Aggregates, 8–14 μm	nr./74–79%	Phenolic compounds, anthocyanins, and cyanidin-3-glucoside	[88]
Apple pectin	*Satureja khuzistanica* Jamzad extract	10:1% *w/w*	3.5 mL/min, 115 °C, nr.	Semi-cubes/Porous, 3 μm	nr./51%	Phenolic compounds	[96]
Apple pectin	*Satureja rechingeri* Jamzad extract	10:1% *w/w*	3.5 mL/min, 115 °C, nr.	Semi-cubes/Porous, 3 μm	nr./38%	Phenolic compounds	[96]
Apple pectin	Vitamin B1	1:0.125% *w/w*	4 mL/min, 120 °C, 50–67 °C	Spherical/Irregular, 0.5–1.3 μm	44%/95%	Vitamin B1	[68]
PG2000	Borage seed oil	10–20:13% *w/w*	10 mL/min, 170 °C, 80 °C	Asymmetrical/Wrinkled, 4–20 μm	nr./87%	Oil	[78]
PG2000	Borage seed oil/Curcumin	10–20:13:0.6% *w/w*/*w*	10 mL/min, 170 °C, 80 °C	Asymmetrical/Wrinkled, 4–20 μm	nr./89%	Curcumin	[78]
PG2000	Borage seed oil/Resveratrol	10–20:13:0.4% *w/w*/*w*	10 mL/min, 170 °C, 80 °C	Asymmetrical/Wrinkled, 4–20 μm	nr./86%	Resveratrol	[78]
PG2000	Borage seed oil/Curcumin/Resveratrol	10–20:13:0.6:0.4% *w/w*/*w/w*	10 mL/min, 170 °C, 80 °C	Asymmetrical/Wrinkled, 4–20 μm	nr./89%	Curcumin and resveratrol	[78]
Brea gum	Corn oil	5–20:10% *w/w*	nr., 150 °C, 60 °C	Semi-spherical/Dents, 0.8–26 μm	nr./34–76%	Oil	[111]
Cashew tree gum	Fish oil	15:nr.% *w/nr*.	0.7 L/h, 180 °C, nr.	Spherical/Smooth holes, 30–63 μm	nr./76%	Oil	[69]
Mesquite gum	Sesame (*Sesamum indica* L.) oil	1–3:1 ratio	nr., 120–160 °C, nr.	Spherical/Wrinkled, nr.	nr./70–90%	Oil	[114]

nr.: not reported; A: achira; C: corn; Carr: carrageenan; CH: chitosan; GA: gum Arabic; C14: myristic acid; C16: palmitic acid; C18: stearic acid; C20: cetoleic acid; HCP: Hi-cap100; M: maize; MD: maltodextrin; Mo: modified; MUFA: monounsaturated fatty acid; P: potato; PUFA: polyunsaturated fatty acid; PG2000: purity gum 2000; R: rice; SA: sodium alginate; SC: sodium caseinate; SFA: saturated fatty acid; SOS: sodium octenylsuccinate; T: taro; W: wheat.

**Table 2:** Encapsulation of bioactive compounds with protein-based wall materials.

Wall Material	Core Material	Concentration (Wall Material: Core Material)	Conditions (Feed Rate, Inlet Air, Outlet Air)	Particles (Shape/Morphology, Particle Size Distribution)	Process Yield/Encapsulation Efficiency	Encapsulated Compounds	References
Gelatin	Brown seaweed (*Saccharina japonica*) extract	10:100% *w/v*	30 mL/min, 230 °C, 105 °C	Spherical/Wrinkled, 15.8 μm	nr./87%	Phenolic acids (gallic acid, chlorogenic acid, gentisic acid, protocatechuic acid, p-hydroxybenzoic acid, vanillic acid, caffeic acid, and syringic acid)	[84]
Gelatin	Thyme (*Thymus serpyllum* L.) extract	5:100% *w/v*	nr., 140 °C, 72–75 °C	Semi-spherical/Wrinkled, 130–223 μm	nr./1–44%	Phenolic compounds, flavonoids, and sugars	[130]
Gelatin	Algal (*Tetraselmis chuii*) biomass	2:1% *w/w*	2.5 mL/min, 150 °C, 60 °C	Spherical/Rough, 1.5–15.3 μm	22–45%/46–78%	Phenolic compounds, β-carotene, and carotenoids	[99]
Gelatin	Ciprofloxacin	0.5–2:1–5% *w/w*	90–308 mL/h, 115–140 °C, nr.	Spherical/Smooth, 2.6–3.7 μm	nr./95–100%	Ciprofloxacin	[131]
Casein	Aloe vera (*Aloe barbadensis*) leaves	4:1% *w/w*	nr., 160 °C, 70 °C	Spherical/Smooth, 2 μm	nr./nr.	Anthraquinone	[132]
Casein	Jaboticaba (*Plinia jaboticaba*) skin extract	1:5 ratio	1 L/h, 190 °C, 81 °C	Spherical/Wrinkled, 3–25 μm	nr./nr.	Phenolic compounds	[133]
Casein	Caffeine	0–1:0–1 ratio	8 mL/min, 100–190 °C, nr.	Irregular/Smooth, 10 μm	20–65%/nr.	Caffeine	[134]
Casein	Ascorbic acid	0.62:0.1% *w/w*	8 mL/min, 100–150 °C, nr.	Spherical/Wrinkled, 3–27 μm	60–80%/20–70%	Ascorbic acid	[135]
Casein	Curcumin	15.5:1% *w/w*	46.5 mL/min, 180 °C, 90 °C	Spherical/Wrinkled, 33 μm	nr./nr.	Curcumin	[136]
SPI	Horseradish leaf (*Armoracia rusticana* L.) juice	20–80:20–80 ratio	0.33 L/h, 120 °C, 80 °C	nr./nr., 7.5–14.0 μm	nr./nr.	Phenolic compounds, rutin, epicatechin, catechin and sinapic acid	[77]
SPI	Horseradish root (*Armoracia rusticana* L.) juice	20–80:20–80 ratio	0.33 L/h, 120 °C, 80 °C	nr./nr., 6.5–6.9 μm	nr./nr.	Phenolic compounds, rutin, epicatechin, catechin and sinapic acid	[77]
C-Zein	α-Tocopherol	1:6 ratio	7–9 mL/min, 110–180 °C, nr.	Irregular/Porous, 0.5–5 μm	44–77%/31–42%	α-Tocopherol	[137]
WP	Capsaicin	10:20% *w/w*	nr., 185 °C, 85 °C	Spherical/Wrinkled, 0.8–8.1 μm	68%/95%	Capsaicin	[138]
WP	Brown seaweed (*Saccharina japonica*) extract	10:100% *w/v*	30 mL/min, 230 °C, 105 °C	Spherical/Wrinkled, 143 μm	nr./87%	Phenolic acids (gallic acid, chlorogenic acid, gentisic acid, protocatechuic acid, p-hydroxybenzoic acid, vanillic acid, caffeic acid, and syringic acid)	[84]
WPI	Tributyrin	3–5:1 ratio	nr., 160 °C, nr.	Spherical/Smooth, 7–11 μm	nr./24–35%	Tributyrin	[139]
WPI	Fresh kale (*Brassica oleracea* L.) leaves extract	5–15:40% *w/v*	12 mL/min, 215 °C, 65 °C	Spherical/Wrinkled, 80 μm	nr./99%	Chlorophyll	[140]
WPI	Mix (paprika-cinnamon oleoresin)	10:1:1%w/ratio/ratio	6 mL/min, 150 °C, 80 °C	Spherical/Porous, 15 μm	43%/84%	Carotenoids	[80]
Milk-PC	Conjugated linoleic acid	1:8% *w/w*	nr., 160 °C, 80 °C	Spherical/Irregular, 10–25 μm	nr./84%	Conjugated linoleic acid	[141]
Rice protein	Propolis extract	1:1% *v/v*	0.4 m^3^/min, 120 °C, 72 °C	Spherical/Rough, nr.	28%/90%	Propolis	[142]
Pea protein	Propolis extract	1:1% *v/v*	0.4 m^3^/min, 120 °C, 72 °C	Spherical/Rough, nr.	41%/90%	Propolis	[142]
Soy protein	Propolis extract	1:1% *v/v*	0.4 m^3^/min, 120 °C, 72 °C	Spherical/Rough, nr.	20%/70%	Propolis	[142]
Ovalbumin	Propolis extract	1:1% *v/v*	0.4 m^3^/min, 120 °C, 72 °C	Spherical/Rough, nr.	53%/73%	Propolis	[142]
HP	Orange essential oil	30% *w/w*	3.75 mL/min, 180 °C, 85 °C	Spherical/Rough, 30–40 μm	36%/89%	D-limonene	[70]

nr.: not reported; C: corn; HP: hydrolyzed protein; SPI: soy protein isolate; WP: whey protein; WPI: whey protein isolate.

## Data Availability

Not applicable.
